# Influence of Epichlorohydrin Concentration on the Physicochemical and Rheological Performance of Lignin/PVA Hydrogels

**DOI:** 10.3390/polym17233223

**Published:** 2025-12-03

**Authors:** Nazish Jabeen, Paula G. Garnero, Rafael Muñoz-Espí, Clara M. Gómez, Mario Culebras

**Affiliations:** Institute of Materials Science (ICMUV), University of Valencia, P.O. Box 22085, E46071 Valencia, Spain; nazish.jabeen@uv.es (N.J.); paula.garnero@uv.es (P.G.G.); rafael.munoz@uv.es (R.M.-E.)

**Keywords:** hydrogel, cross-linking, epichlorohydrin, rheological properties, gelation kinetics

## Abstract

This study investigates the influence of epichlorohydrin (EPCH) concentration on the rheological, mechanical, and swelling properties of lignin/PVA hydrogels. Hydrogels were prepared with EPCH concentrations ranging from 2.5% to 7.5%, and their viscoelastic properties were characterized through oscillatory strain and frequency sweep rheology. Increasing the EPCH concentration led to a substantial rise in mechanical stiffness, with the compressive modulus increasing from 21 kPa (2.5%) to 275 kPa (7.5%), accompanied by a marked reduction in swelling capacity from 460% to 190%. This behavior is attributed to the formation of a denser and more interconnected network structure with increasing cross-linking density. Furthermore, a strong correlation was observed between EPCH concentration and gelation kinetics, with higher concentrations generally leading to faster gelation times. In all formulations, gel time consistently decreased as the temperature increased from 10 to 50 °C. The optimal EPCH concentration for achieving a balance between mechanical properties and processability was determined to be 3.5%. At this concentration, the hydrogels exhibited a favorable combination of mechanical strength, shape recovery, and processability, while maintaining desirable swelling behavior. These findings provide valuable insights into the critical role of cross-linking density in determining the physicochemical properties of lignin/PVA hydrogels, paving the way for the development of these bio-based materials with tailored properties for diverse applications.

## 1. Introduction

Hydrogels are remarkable soft materials composed of a three-dimensional network of polymer chains interconnected through physical or chemical bonds. These intricate structures exhibit exceptional water absorption capacity, often exceeding 99.9% of their weight [1,2]. This highly porous network confers exceptional swelling properties to hydrogels, rendering them versatile candidates for a wide range of applications [3]. In recent years, hydrogels have emerged as a focus of intense research in diverse fields, including wastewater treatment [4], drug delivery systems [5,6], wound healing [7], wearable electronics [8,9], and supercapacitors [10]. Their widespread use can be attributed to their beneficial properties, such as non-toxicity, excellent biocompatibility, exceptional water absorption capacity, and the ability to self-assemble. Among the diverse array of hydrogels, poly(vinyl alcohol) (PVA) stands out as a prominent water-soluble polymer. The presence of numerous hydroxyl groups within each repeating unit of PVA facilitates the formation of chemically or physically cross-linked gel networks [11]. However, conventional hydrogels were generally brittle and hard to recover, which limited the application under harsh rheology conditions [12]. To extend their application areas, significant research efforts have been directed towards synthesizing structurally diverse PVA-based hydrogels by incorporating other polymers through cross-linking strategies. These modifications aim to enhance the mechanical and rheological properties of pure PVA hydrogels, overcoming their inherent brittleness and limited recoverability [13]. Biomass lignin could be used to improve the rheological properties of the PVA hydrogel [14,15].

Lignin, a renewable biopolymer derived from lignocellulosic biomass, represents a significant source of aromatic compounds. Its unique characteristics, including low cost, environmental friendliness, and biodegradability, position lignin as a promising candidate for reinforcing polymeric materials [16]. Notably, lignin possesses an intricate aromatic structure adorned with diverse surface functional groups, enabling it to act as a spacer within the polymer matrix. This can facilitate the formation of a finely porous network, significantly enhancing the mechanical and rheological properties of the resulting hydrogels [17]. The gelation process, which involves the gradual transformation of a liquid precursor into a solid gel, plays a crucial role in determining the final properties of the hydrogel. Understanding and controlling the factors that influence gelation kinetics, such as the concentration and reactivity of the cross-linking agent, are essential for tailoring hydrogel properties [18,19].

This study focused on investigating the influence of epichlorohydrin (EPCH) concentration on the synthesis and characterization of lignin/PVA hydrogels. By systematically varying the EPCH concentration, we aimed to explore its impact on the rheological, mechanical, and morphological properties of the resulting hydrogels, including their swelling behavior, compressibility, and overall structural integrity. These findings will contribute to a deeper understanding of the interplay between cross-linking density and hydrogel properties, paving the way for the development of high-performance lignin-based hydrogels with tailored properties for a wide range of applications.

## 2. Materials and Methods

### 2.1. Materials

Kraft lignin *M*_w_ = 3715 g/mol, (*Ð* ≈ 4), was obtained from ENCE Energía y Celulosa SA (Madrid, Spain). Sodium hydroxide (NaOH) pellets with 98% purity, epichlorohydrin (EPCH) with 99% purity, and poly(vinyl alcohol) (PVA) with 99% hydrolyzed purity (Mw of 85,000–124,000 g/mol) were procured from Sigma-Aldrich (Barcelona, Spain).

### 2.2. Synthesis of Lignin/PVA Hydrogel

**Figure 1:**
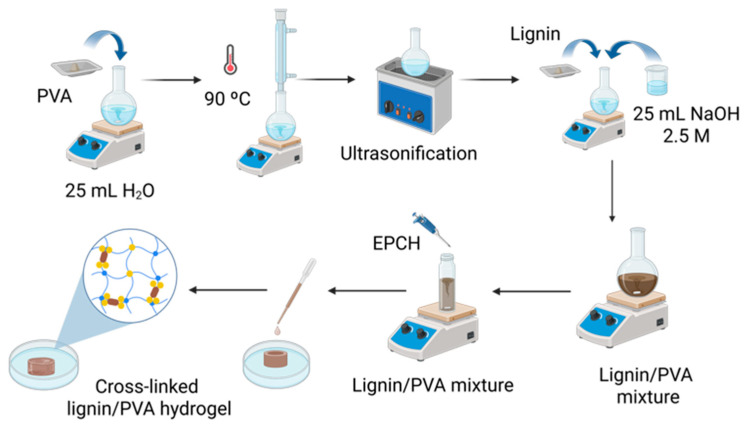
Schematic representation of lignin/PVA hydrogel synthesis.

To prepare the hydrogel, 4 g of PVA was gradually added to 25 mL of deionized water under constant stirring at room temperature to ensure the formation of a homogeneous PVA solution, as illustrated in Figure 1. The solution was vigorously stirred at 90 °C for 1 h to achieve complete dissolution of PVA. Afterwards, the solution was cooled to room temperature and ultrasonicated for 5 min to remove any entrapped air bubbles. Subsequently, 25 mL of NaOH solution was added dropwise to the PVA solution under continuous stirring, followed by the gradual incorporation of 4.5 g of lignin. The resulting mixture was magnetically stirred at room temperature for 5 h to obtain a uniform lignin/PVA blend. Subsequently, specific volumes of epichlorohydrin (EPCH), as detailed in Table 1, were added to 5 mL of the lignin mixture and stirred thoroughly for 30 min. The resulting solution was then poured into cylindrical molds with dimensions of 12 mm in diameter and 6 mm in thickness. After 24 h of curing at room temperature, the cross-linked hydrogel was carefully removed from the molds, yielding the final lignin/PVA hydrogel samples.

### 2.3. Characterization Techniques

Lignin/PVA hydrogels were investigated for functional group analysis using FTIR spectroscopy. Measurements were taken in transmittance mode on an Agilent Cary 630 FTIR spectrophotometer (Agilent Technologies, Santa Clara, CA, USA) across a wavenumber range of 500 to 4000 cm^−1^ with 32 scanning times. The thermal behavior of the hydrogel samples (5–10 mg) was examined using two complementary techniques: differential scanning calorimetry (DSC) and thermogravimetric analysis (TGA). For the DSC analysis (TA Instruments DSC Q20 instrument, New Castle, DE, USA), the sample was heated from 0 to 220 °C at a rate of 10 °C/min under a nitrogen purge gas flow. Similarly, during TGA analysis (TA Instruments TGA 550 instrument, New Castle, DE, USA), the sample was subjected to a temperature program ranging from room temperature to 900 °C at 10 °C/min under a nitrogen flow rate of 10 mL/min.

### 2.4. Rheological Measurements

Rheological measurements were conducted to characterize the viscoelastic properties of the hydrogels, using a Kinexus^®^ Prime lab+ rheometer equipped with a 40 mm plate-plate geometry (Netzsch, Barcelona, Spain). Initially, the linear viscoelastic region (LVER) was determined by performing strain sweeps on lignin/PVA samples at 1 Hz. Within the LVER, the material exhibits linear viscoelastic behavior, where the stress response is directly proportional to the strain and the material reversibly deforms upon load removal, adhering to Hooke’s law [20,21]. This procedure was conducted at all desired working temperatures. Subsequently, time-dependent oscillatory rheological measurements were conducted to monitor the evolution of storage modulus (G′) and loss modulus (G″) during the cross-linking reaction. A specific volume of EPCH, as detailed in Table S1, was added to 5 mL of the lignin/PVA mixture and vigorously stirred for 3 min. The hydrogel precursor solution was then transferred to the rheometer, and the selected temperature was stabilized for 10 min before commencing measurements. Time-dependent measurements of G′ and G″ were recorded for 20.5 h at a constant frequency of 1 Hz, ensuring the applied strain remained within the LVER. Finally, the obtained data were plotted, and the gel point was determined by identifying the point of intersection between the G′ and G″ curves [22].

### 2.5. Swelling Behavior

To investigate the swelling behavior, the lignin/PVA hydrogels were thoroughly washed with deionized water to remove any unreacted reagents and impurities. Subsequently, the washed hydrogels were dried in a vacuum oven at 35 °C for 48 h. The dried hydrogels were accurately weighed using an analytical balance. Following this, dried hydrogels were immersed in deionized water, and their weight was recorded after 30 min intervals for the first 5 h, followed by measurements every 24 h for the next 48 h. The swelling capacity was calculated for each measurement using Equation (1) and plotted as a function of time.

(1) $$\mathrm{Swelling}\;\mathrm{capacity}\;(\%)=\frac{\left(m_{t}-m_{0}\right)}{m_{0}}\times 100$$

where *m*_0_ denotes the mass of the dried hydrogels and *m_t_* is the mass of the swollen hydrogels at different times. The swelling procedure was performed in triplicate for each EPCH concentration.

### 2.6. Compression Test

Compression tests were conducted using an Instron 5582 universal testing machine equipped with 5 cm diameter plates and a 100 kN load cell. A constant strain rate of 2 mm/min was applied to all lignin/PVA hydrogel samples. Three replicate samples were analyzed for each cross-linker concentration.

## 3. Results and Discussion

**Figure 2:**
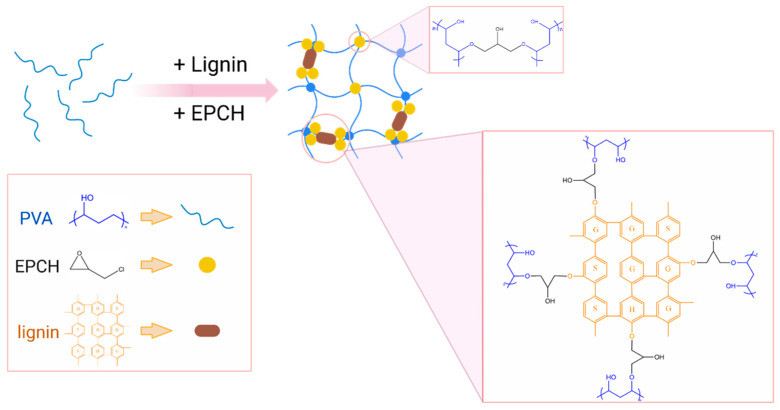
The structural mechanism of the lignin/PVA hydrogel using EPCH as a cross-linker (inspired by ref. [25]).

Figure 1 illustrates the schematic representation of the lignin/PVA hydrogel synthesis process. The process commences with the dissolution of PVA in an aqueous NaOH solution, followed by the addition of Kraft lignin and subsequent overnight stirring to ensure complete homogenization [23]. EPCH is utilized as a cross-linking agent to cross-link polymeric molecules. The alkaline environment promotes the deprotonation of lignin, enhancing its reactivity and facilitating the formation of cross-linked networks [24]. This involves the formation of ether bonds between the phenolic hydroxyl groups of lignin, the aliphatic hydroxyl groups of PVA, and the epoxy groups of EPCH (see Figure 2). As the reaction proceeds, HCl is eliminated from one end of EPCH, resulting in the generation of additional epoxy groups [25]. Furthermore, strong intermolecular hydrogen bonding between PVA and the hydroxyl groups of lignin contributes to the formation of a dense and interconnected network. However, lignin/PVA hydrogels synthesized with higher EPCH concentrations (5.0%, 6.0%, and 7.5%) exhibited significantly enhanced stiffness compared to those prepared with lower concentrations (2.5% and 3.5%). This observation highlights the crucial role of cross-linking density in determining the mechanical properties of the resulting hydrogels.

**Figure 3:**
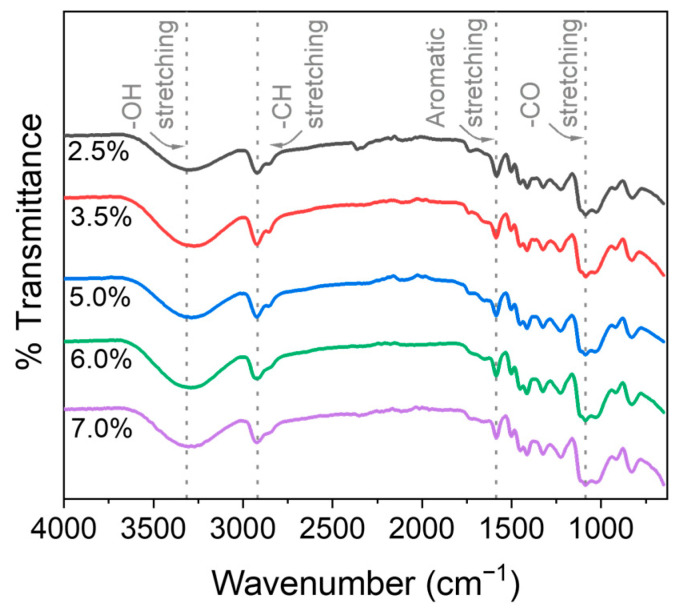
FTIR spectra of the lignin/PVA hydrogels with various EPCH concentrations.

Figure 3 illustrates the FTIR spectra of lignin/PVA hydrogels, providing valuable insights into the effects of chemical cross-linker concentration on the chemical structure and degree of cross-linking of the hydrogels. All hydrogels exhibited similar FTIR spectra; the intensity of some prominent peaks, however, varied with the degree of cross-linking. The intensity of the broad peak around 3300 cm^−1^, associated with –OH stretching vibrations in lignin/PVA hydrogels, decreased with increasing EPCH concentration [23,24]. This reduction is attributed to the consumption of available hydroxyl groups for covalent bonding at higher cross-linker concentrations, resulting in a highly cross-linked hydrogel network and limited availability of –OH functional sites [26]. Peaks corresponding to –CH_3_ and –CH_2_ stretching between 3150 and 2840 cm^−1^ are attributed to the aromatic structure and carbonyl functional groups present on the surface of the lignin-derived hydrogels [25]. An increase in the intensity of the C–O stretching band near 1090 cm^−1^ confirmed the formation of new ether (C–O–C) linkages within the polymer network as cross-linking increases. However, the C–O stretching band exhibited only a very slight increase with increasing EPCH concentration, which may reasonably be attributed to baseline variation rather than a significant enhancement. The aromatic skeletal vibrations at 1586 and 1451 cm^−1^ remained at consistent wavenumbers across all cross-linker concentrations, showing no significant shift [27]. However, changes in the shape of these bands provide evidence of structural modifications in the lignin framework associated with successful cross-linking between lignin and PVA.

**Figure 4:**
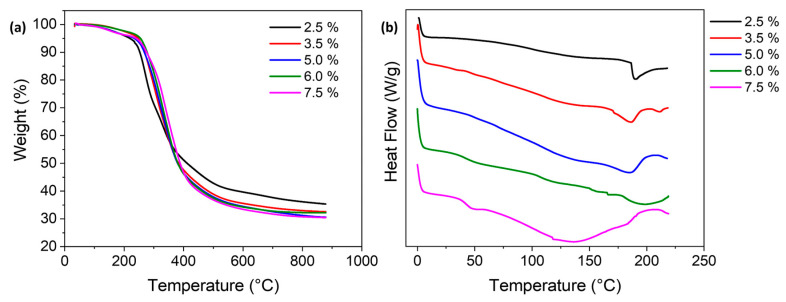
(**a**) TGA and (**b**) DSC thermograms of lignin/PVA hydrogels with various EPCH concentrations.

To further evaluate the thermal properties of the lignin/PVA hydrogels, thermogravimetric analysis (TGA) was performed, varying EPCH concentrations under nitrogen atmosphere. As shown in Figure 4a, thermograms displayed three distinct weight loss stages for all hydrogel samples, beginning with a reduction up to approximately 150 °C due to the evaporation of physically and chemically bound water, indicating similar initial moisture content across formulations [23]. The primary degradation phase occurred between 200 and 400 °C, corresponding to decomposition of the PVA backbone and lignin structures. Specifically, the onset degradation temperature (*T*_onset_) increased from ~220 °C (2.5% EPCH) to ~230 °C (3.5%), and remained near ~235 °C for 5.0%, 6.0%, and 7.5% EPCH. A similar trend was observed for the temperature at 20% weight loss (*T*_20%_), which rose from ~275 °C (2.5%) to ~293 °C (3.5%), ~300 °C (5.0%), ~308 °C (6.0%), and ~316 °C (7.5%). Likewise, the temperature at 40% weight loss (*T*_40%_) shifts upward from ~340 °C at 2.5% EPCH to ~345 °C (3.5%), ~350 °C (5.0%), ~353 °C (6.0%), and ~362 °C (7.5%). These progressive increases confirm that higher EPCH concentration shifts the onset of thermal degradation to higher temperatures, demonstrating improved thermal stability due to greater cross-linking density, which reinforces the hydrogel network and raises its resistance to thermal breakdown. In the final degradation stage above 400 °C, samples with higher EPCH concentrations exhibited slightly lower residual mass, decreasing from ~36% (2.5% EPCH) to ~33% (3.5%), ~31% (5.0%), ~32% (6.0%), and ~30% (7.5%) at 800 °C. This behavior is attributed to the enhanced cross-linking density and the inherently aromatic structure of lignin, which promotes the formation of a stable carbonaceous residue during pyrolysis [28]. These results demonstrate that increased EPCH cross-linking improves the thermal stability of lignin/PVA hydrogels and leads to a thermally resistant polymer network.

The possible thermal transitions of the hydrogels were further examined using differential scanning calorimetry (DSC). As shown in Figure 4b, these hydrogels exhibited a clear correlation between EPCH cross-linker concentration and network rigidity, primarily reflected in the glass transition temperature (*T*_g_). The *T*_g_, determined from the midpoint of the baseline shift in the DSC thermogram, systematically increases with rising cross-linker concentration [23]. The least cross-linked hydrogel (2.5%) showed a *T*_g_ of 105.8 °C, which increased to 111.4 °C for 3.5%, 118.9 °C for 5.0%, and 124.6 °C for 6.0%. The highest *T*_g_ value, 132.2 °C, was recorded for the 7.5% sample, confirming that stronger covalent cross-linking effectively restricts polymer chain mobility and enhances thermal stability. Moreover, the DSC thermograms revealed a gradual suppression of the PVA melting endotherm, typically observed between 170 °C and 210 °C, with increasing EPCH concentration. This suppression indicates reduced crystallinity due to restricted molecular rearrangement, resulting in the formation of a more amorphous polymer network at higher cross-linking levels.

### 3.1. Linear Viscoelastic Region (LVER)

**Figure 5:**
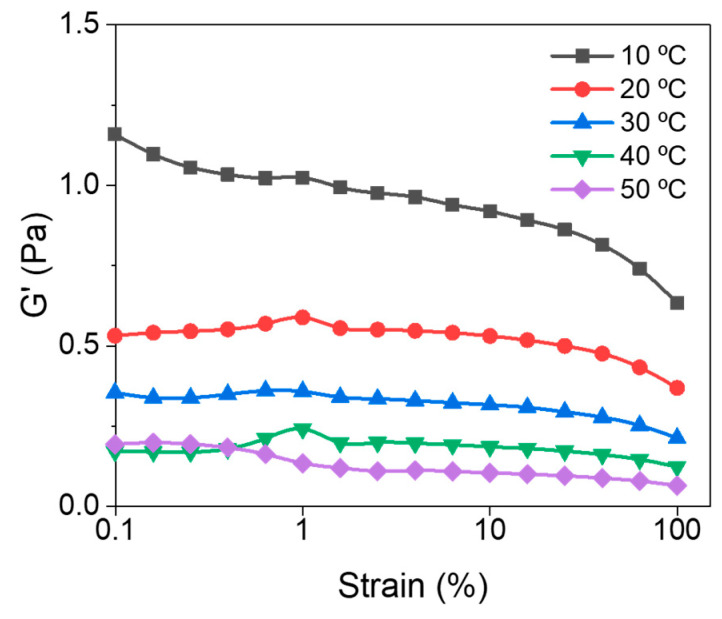
Storage moduli of lignin/PVA mixture as a function of strain at various temperatures.

Figure 5 depicts the results of strain sweep experiments conducted to determine the Linear Viscoelastic Region (LVER) by plotting the storage modulus (G’) as a function of strain at various temperatures. Based on these results, a conservative strain of 2.5% was selected within the LVER for all subsequent oscillatory rheology experiments. This region guarantees a linear response regime where the stress is directly proportional to the strain, and all deformations are fully reversible. This approach is crucial for accurately characterizing the viscoelastic properties of the hydrogel while minimizing the risk of irreversible structural changes that can occur at higher strain amplitudes [20,21].

**Figure 6:**
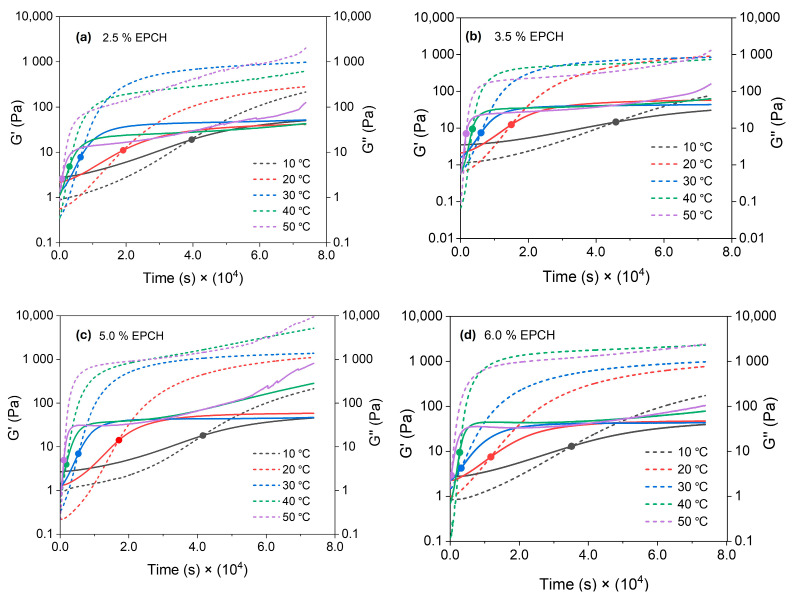
Loss (G″, solid line) and storage (G′, pointed line) moduli evolution at different cross-linking times for different EPCH concentrations at different temperatures. (**a**) 2.5%, (**b**) 3.5%, (**c**) 5.0%, and (**d**) 6.0%. Y-axes are in logarithmic scale.

The rheological behavior of the hydrogels was investigated by monitoring the evolution of storage modulus (G′) and loss modulus (G″) over time, as depicted in Figure 6a–d. The crossover point, where G′ and G″ intersect, signifies the transition from a predominantly viscous to an elastic state and thus provides an indication of the gelation time for each formulation [19,29,30,31]. Notably, the rheological data for the 7.5% EPCH hydrogels exhibited significant inconsistencies and were therefore excluded from further analysis. This anomalous behavior is likely attributed to EPCH oversaturation within the system, leading to unpredictable and inconsistent cross-linking kinetics. This hypothesis is supported by the color difference observed in the 7.5% EPCH hydrogels compared to those with lower EPCH concentrations. EPCH, being an organic compound, can lead to system saturation at higher concentrations. This saturation likely reduces lignin solubility and compromises the overall homogeneity of the mixture. Consequently, these factors contribute to the inconsistent and unpredictable gelation times observed in the lignin/PVA samples with elevated EPCH content.

**Figure 7:**
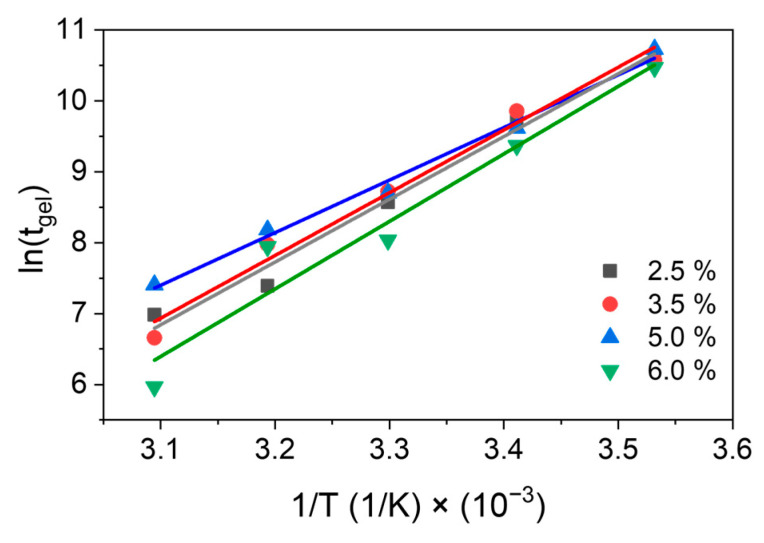
Natural logarithm of gel time as a function of the inverse of temperature for 2.5%, 3.5%, 5.0%, and 6.0% EPCH concentrations.

The gel times obtained for the 2.5%, 3.5%, 5.0%, and 6.0% EPCH hydrogels followed an Arrhenius-type equation [32], as depicted in Figure 7, by plotting ln(*t*_gel_) as a function of 1/T (T= 10, 20, 30, 40 and 50 °C).

**Figure 8:**
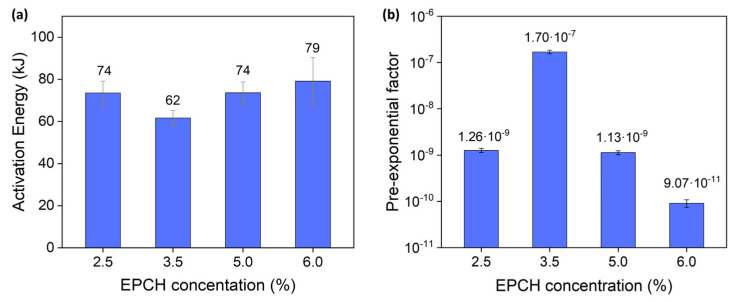
(**a**) Activation energy at different EPCH concentrations. (**b**) Pre-exponential factor at different EPCH concentration (y-axis in logarithmic scale). All the data were collected from linear Arrhenius plot fittings.

Pre-exponential factors and activation energies were calculated according to equation:

(2) $$\ln\left(t_{gel}\right)=\frac{E_{gel}}{R}\;(\frac{1}{T})+\ln(A)$$

where *t*_gel_ is the crossover time in seconds, *E*_gel_ is the activation energy of the gelation process in joules (J), *R* is the universal constant, which has a value of 8.3144621 J·K^−1^·mol^−1^, *T* refers to the measurement temperature, and *A* is the pre-exponential factor, also known as the frequency factor. The pre-exponential factor is specific to each reaction and indicates the frequency of collisions between reactant molecules with the appropriate orientation. The fitting parameters are summarized in Table 2, and the results for activation energy and the pre-exponential factor are illustrated in Figure 8a,b [29,30,32].

Analysis of the pre-exponential factor revealed a peak at 3.5% EPCH concentration, indicating optimal conditions for effective molecular collisions necessary for cross-linking. This suggests that at this specific concentration, the system achieves optimal conditions for cross-linking, maximizing the efficiency of molecular interactions. However, a notable decrease in the pre-exponential factor was observed at higher EPCH concentrations (5.0% and 6.0%), signifying a reduction in the frequency of successful molecular interactions. This decline is likely attributable to saturation effects within the system, which can disrupt the uniformity of network formation and result in less efficient cross-linking [17].

These findings are further supported by the observed trends in activation energy. A lower activation energy of 62 kJ/mol was observed at an EPCH concentration of 3.5%, suggesting that gelation at this concentration occurs more readily, requiring less energy input. This indicates a more favorable environment for cross-linking reactions. Conversely, higher EPCH concentrations (5.0% and 6.0%) resulted in increased activation energies, reaching 74 kJ/mol and 79 kJ/mol, respectively. This trend suggests that higher EPCH concentrations result in a more rigid and less reactive system, requiring a greater energy input to initiate gelation. This increased energy barrier likely reflects increased resistance to cross-linking, potentially attributed to oversaturation effects and reduced lignin solubility at these higher concentrations. Additionally, 2.5% does not appear to be high enough for the optimization of the cross-linking reaction [33,34]. Collectively, these results strongly suggest that an EPCH concentration of 3.5% represents the optimal condition for cross-linking lignin/PVA hydrogels, effectively balancing efficient molecular interactions with lower energy requirements for gelation. Increasing the EPCH concentration to 5.0% and 6.0% results in system oversaturation, leading to reduced lignin solubility and compromised homogeneity. This, in turn, increases the energy barrier for gelation and diminishes the consistency of the cross-linking process. The results obtained were further supported by the observed mechanical properties and inconsistent behavior of 7.5% EPCH in hydrogels, indicative of significant changes in system uniformity due to oversaturation effects.

**Figure 9:**
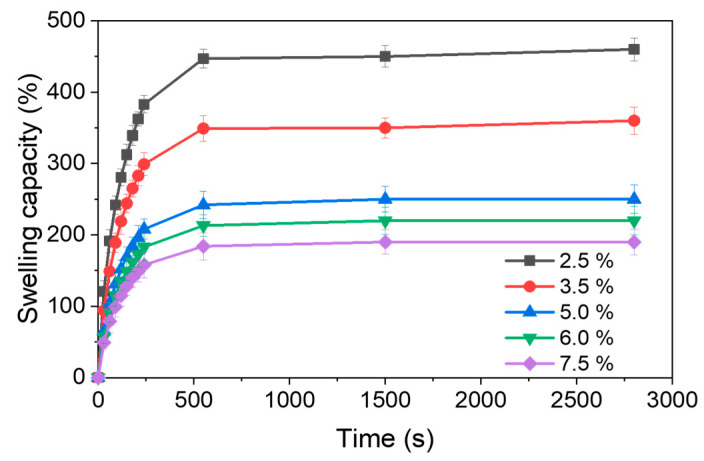
Swelling capacity as a function of time for lignin/PVA hydrogels at different EPCH concentrations.

Figure 9 illustrates the swelling behavior of lignin/PVA hydrogels as a function of time for various EPCH concentrations. The maximum swelling capacity was observed in the hydrogel prepared with 2.5% EPCH, reaching 460%. A clear inverse correlation between EPCH concentration and swelling capacity was observed. As the EPCH concentration increased to 3.5%, 5.0%, 6.0%, and 7.5%, the maximum swelling capacity significantly decreased to 360%, 250%, 220%, and 190%, respectively. This reduction in swelling capacity can be attributed to the increased cross-linking density within the hydrogel network [24]. Higher EPCH concentrations lead to the formation of a denser and more interconnected network structure, which restricts water diffusion and limits water uptake [25].

### 3.2. Mechanical Properties

**Figure 10:**
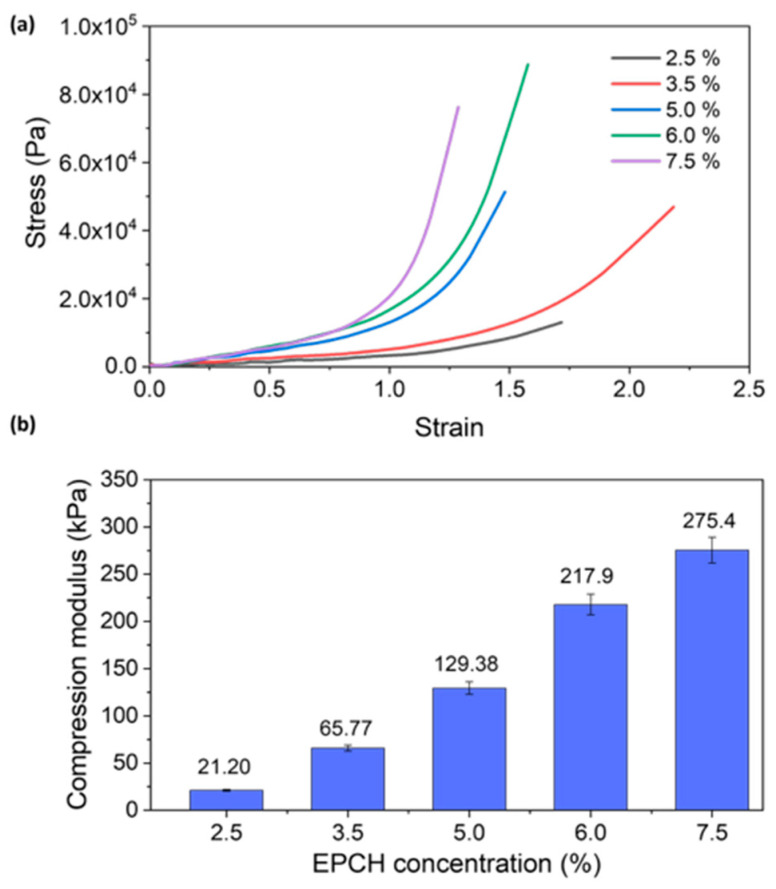
(**a**) Compression testing stress–strain curves. (**b**) Compressive modulus of lignin/PVA hydrogels as a function of EPCH concentrations (2.5%, 3.5%, 5.0%, 6.0%, and 7.5%).

The mechanical properties of the hydrogels were evaluated through compression tests. Figure 10a presents the stress–strain curves for hydrogels produced at room temperature, with each data point representing the average of three independent replicates. A clear trend of increasing mechanical strength was observed with an increasing cross-linker ratio from 2.5 to 7.5%. The slope of the linear region within each stress–strain curve was determined to calculate the compression modulus of each hydrogel, as depicted in Figure 10b. The compression modulus exhibited a significant increase with increasing EPCH concentration, rising from 21.20 kPa at 2.5% to 275.4 kPa at 7.5% EPCH cross-linker, demonstrating a substantial enhancement in the mechanical properties of the hydrogels [17,34]. This trend directly correlates with the increasing density of the cross-linked polymer network. As the EPCH concentration increases, the number of covalent bonds formed between polymer chains increases, leading to a denser and more interconnected network structure. This denser network restricts the mobility of polymer chains, thereby enhancing the hydrogel resistance to deformation and increasing its compressive strength [35].

Furthermore, the compressive stress–strain analysis revealed notable differences in shape recovery. Hydrogels prepared with lower EPCH concentrations (2.5% and 3.5%) exhibited limited shape recovery after compression, indicating a less robust network structure [33]. At these lower concentrations, the formation of covalent bonds was less extensive, resulting in a more loosely interconnected network. This less-constrained network allows for greater polymer chain mobility, leading to significant deformation under compressive stress and subsequent inability to fully recover its original shape.

**Figure 11:**
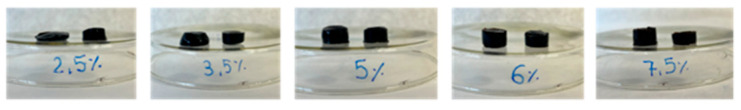
Compressed hydrogels (**left**) next to uncompressed ones of the same ratio (**right**).

In contrast, hydrogels prepared with higher EPCH concentrations (5.0%, 6.0%, and 7.5%) demonstrated excellent shape recovery, indicating a significant enhancement in their resilience. The higher degree of cross-linking within these hydrogels effectively restricts polymer chain mobility, enabling the network to store elastic energy during compression. Upon release of the compressive force, this stored energy drives the network back to its original configuration, demonstrating superior shape memory properties. This observation is demonstrated in Figure 11, where hydrogels with higher EPCH concentrations (5.0%, 6.0%, and 7.5%) readily recovered their original shape after compression, while those with lower concentrations exhibited significant deformation.

## 4. Conclusions

This study comprehensively investigated the influence of epichlorohydrin (EPCH) concentration on the physicochemical properties of lignin/PVA hydrogels. Rheological analysis revealed a strong correlation between EPCH concentration and gelation kinetics, demonstrating that higher concentrations generally resulted in faster gelation times. Furthermore, a significant enhancement in mechanical properties, including increased compressive modulus and improved shape recovery, was observed with increasing EPCH concentration. This enhancement can be directly attributed to the formation of a denser and more interconnected network structure resulting from the increased cross-linking density. The optimal EPCH concentration was determined to be 3.5%, exhibiting a favorable balance between mechanical strength, shape recovery, and processability. Higher EPCH concentrations, while enhancing mechanical properties, may lead to undesirable effects such as system saturation and reduced lignin solubility, potentially hindering processability and impacting the overall homogeneity of the hydrogel network. These findings provide valuable insights into the critical role of cross-linking density in determining the physicochemical properties of lignin/PVA hydrogels. This study can be effectively utilized to optimize the synthesis process and tailor the properties of these bio-based materials for specific applications, including tissue engineering, drug delivery, energy harvesting applications. Future research should explore the potential of incorporating other biopolymers or modifying the cross-linking chemistry to enhance the properties further and broaden the applications of these promising materials.

## Figures and Tables

**Table 1:** Epichlorohydrin volumes for hydrogel preparations.

EPCH (*v*/*v*%)	Total Volume PVA/Lignin/EPCH Mixture (mL)	Volume PVA/Lignin Mixture (mL)	Volume EPCH (μL)
2.5	5	4.875	125
3.5	5	4.82	175
5.0	5	4.75	250
6.0	5	4.70	300
7.5	5	4.63	375

**Table 2:** Arrhenius linear fit parameters.

EPCH (% *v*/*v*)	ln(*A*)	*E*_gel_/*R* (mol/K)	*R* ^2^
2.5	−20 ± 2	8847 ± 700	0.98546
3.5	−15.6 ± 1.4	7415 ± 400	0.98303
5.0	−21 ± 2	8853 ± 600	0.94400
6.0	−23 ± 4	9522 ± 1300	0.99035

## Data Availability

The original contributions presented in this study are included in the article/Supplementary Materials. Further inquiries can be directed to the corresponding authors.

## Supplementary Materials

The following supporting information can be downloaded at: https://www.mdpi.com/article/10.3390/polym17233223/s1, Table S1. Epichlorohydrin volumes for hydrogel preparations; Table S2. Arrhenius linear fit parameters.
