# Supplementary material for: Influence of Epichlorohydrin Concentration on the Physicochemical and Rheological Performance of Lignin/PVA Hydrogels
Source: Polymers (Basel). 2025 Dec 3;17(23):3223. doi: 10.3390/polym17233223 (PMC12693750; doi:10.3390/polym17233223)
Supplement: Supplementary file 1 [file polymers-17-03223-s001.zip › polymers-3987851-supplementary.pdf]

Influence of Epichlorohydrin Concentration on the Physicochemical and Rheological Performance of Lignin/PVA Hydrogels

Nazish Jabeen, Paula G. Garnero, Rafael Muñoz-Espí, Clara M. Gómez * and Mario Culebras *

Institute of Materials Science (ICMUV), University of Valencia, P.O. Box 22085, E46071 Valencia, Spain; nazish.jabeen@uv.es (N.J.); paula.garnero@uv.es (P.G.G.); rafael.munoz@uv.es (R.M.-E.)
* Correspondence: clara.gomez@uv.es (C.M.G.); mario.culebras@uv.es (M.C.)

Supplementary information

Synthesis of lignin/PVA hydrogel

To prepare the hydrogel, 4 g of PVA was gradually added to 25 mL of deionized water under constant stirring at room temperature to ensure the formation of a homogeneous PVA solution, as illustrated in Fig. 1. The solution was vigorously stirred at 90 °C for 1 h to achieve complete dissolution of PVA. Afterwards, the solution was cooled to room temperature and ultrasonicated for 5 min to remove any entrapped air bubbles. Subsequently, 25 mL of a 2.5 M NaOH solution was added dropwise to the PVA solution under continuous stirring, followed by the gradual incorporation of significant amount of lignin. The resulting mixture was magnetically stirred at room temperature for 5 h to obtain a uniform lignin/PVA blend. Subsequently, specific volumes of epichlorohydrin (EPCH), as detailed in Table S1, were added to 5 mL of the lignin mixture and stirred thoroughly for 30 min. The resulting solution was then poured into cylindrical molds with dimensions of 12 mm in diameter and 6 mm in thickness. After 24 h of curing, the cross-linked hydrogel was carefully removed from the molds, yielding the final lignin/PVA hydrogel samples.

Table S1. Epichlorohydrin volumes for hydrogel preparations.

EPCH (% v/v)	Volume PVA/lignin/PVA mixture (mL)	Volume EPCH (μL)
2.5	5	125
3.5	5	175
5.0	5	250
6.0	5	300
7.5	5	375

Pre-exponential factors and activation energies were calculated according to following equation:

$$\ln(t_{\text{gel}}) = \frac{E_{\text{gel}}}{R} \cdot \left(\frac{1}{T}\right) + \ln(A)$$

where t_{gel} is the crossover time in seconds, E_{gel} is the activation energy of the gelation process in joules (J), R is the universal constant (8.3144621 $J \cdot K^{-1} \cdot mol^{-1}$), T refers to the measurement temperature, and A is the pre-exponential factor, also known as the frequency factor. The pre-exponential factor is specific to each reaction and indicates the frequency of collisions between reactant molecules with the appropriate orientation. The fitting parameters are summarized in Table S2.

Table S2. Arrhenius linear fit parameters.

EPCH (% v/v)	ln(A)	E_{gel}/R (mol/K)	R^2
2.5	−20±2	8847±700	0.98546
3.5	−15.6±1.4	7415±400	0.98303
5.0	−21±2	8853±600	0.94400
6.0	−23±4	9522±1300	0.99035
